# Direct Oral Anticoagulants (DOAC): Are We Ready for a Pharmacogenetic Approach?

**DOI:** 10.3390/jpm12010017

**Published:** 2021-12-29

**Authors:** Raffaele Palmirotta

**Affiliations:** Interdisciplinary Department of Medicine, School of Medicine, University of Bari Aldo Moro, 70124 Bari, Italy; raffaele.palmirotta@uniba.it

Anticoagulants play an important role in reducing complications and mortality associated with thromboembolic disorders, and anticoagulant therapy has been progressively enriched over the last few years with numerous new options.

For some time, vitamin K antagonists (VKAs) have been the main drugs used for long-term oral anticoagulant therapy, though they present many limitations such as considerable management difficulties, a considerable percentage of hemorrhagic events, drug and food interactions, and requiring the frequent monitoring of laboratory parameters [1]. Due to the significant limitations of VKAs, over the past decade, pharmacological research has led to the development of new direct oral anticoagulants (DOACs) focusing on molecules able to block individual and specific steps of the coagulative cascade, particularly thrombin (factor IIa) or the Stuart factor (Xa), that are involved in the final common pathway of the coagulative cascade [2,3].

The direct inhibitors of thrombin (dabigatran) act by modulating the transformation of fibrinogen into fibrin and inhibiting the thrombin-mediated activation of factors V, VII, XI, and XII with an anticoagulant effect. In addition, the thrombin blockage also inhibits some effects mediated by receptor-binding such as platelet aggregation. Factor Xa inhibitors (rivaroxaban, apixaban, edoxaban, and betrixaban) act by reducing thrombin formation upstream but do not block the circulating thrombin whose traces may participate in hemostasis by giving to the therapeutic strategy a higher safety profile on hemorrhagic risk [1,4]. New DOACs have been developed with the aim of providing a higher efficacy, or at least comparable to that of VKAs, but with a predictable pharmacokinetic profile, a low intra- and inter-individual variability, reduced hemorrhagic risk, and a wider therapeutic index, all characteristics that would eliminate the needing for routine laboratory monitoring [5,6].

In this regard, a recent observational study of 25,551 patients in the GARFIELD-AF real-world registry showed that all-cause mortality and bleeding rates were significantly lower in patients treated with DOACs than in those treated with VKAs [3].

As widely demonstrated by recent studies, the individual response to drugs is a multifactorial condition, depending on the interaction between environmental and genetic factors. An insufficient response to drug treatment can cause a partial or total failure of the current therapy, while an excessive response can cause serious and sometimes fatal side effects and adverse reactions [7].

Regarding oral VKAs, the influence of genetic factors in response to therapeutic treatment has been demonstrated by numerous literature studies. For example, significant correlations between genotype and response to treatment have been found for single-nucleotide polymorphisms (SNPs) of *VKORC1* and *CYP2C9* genes that significantly influence the response to warfarin treatment, as reported in the guidelines of the Clinical Pharmacogenetics Implementation Consortium (CPIC) [8]. On the contrary, the pharmacogenetic aspects are still poorly understood and still under investigation, despite the increasing use of DOACs and an ascertained variability in response to therapy with these drugs.

A few years after the introduction of DOACs into clinical practice, several pharmacogenetic studies have been reported in the literature, and consequently, some review articles have been produced with the aim to summarize the state-of-art and highlight the correlations between genetic variants and this new class of drugs [2,6,9,10].

In 2019, Kanuri S.H. and Kreutz R.P. first described in an extensive/exhaustive review the pharmacogenetic studies carried out over the past decade of the DOACs dabigatran, rivaroxaban, apixaban, and edoxaban. The authors described, for each drug, the approved indications, mechanism of action, pharmacokinetics, pharmacodynamic, side effects, antidotes, drug-drug interactions, and particularly, the relationship between SNPs of common genetic variants and plasma drug levels [6]. In particular, the polymorphisms of *ABCB1* and *CES1* genes were highlighted. The gene *ABCB1* (ATP-Binding Cassette, Subfamily B, Member 1), also known as *MDR1* (Multidrug Resistance Protein 1), encodes for a transmembrane ATP-dependent transporter P-glycoprotein (p-gp) capable of excreting a wide range of drugs; xenobiotic compounds from cells [11]. *CES1* (Carboxylesterase 1) encode for a liver carboxylesterase capable of hydrolyzing various xenobiotics and endogenous substrates mainly involved in drug metabolism and the detoxification of harmful chemicals [12]. Assessing across studies the effects of polymorphisms on drug plasma levels, the authors concluded that *ABCB1* and *CES1* SNPs, sometimes even combined into haplotypes, contribute to altered peak and trough levels of dabigatran, and several *ABCB1* SNPs are implicated in altered plasma levels of rivaroxaban and apixaban. In addition, the authors provided a useful table showing allele frequency and genotype distributions in different ethnic groups of common variants of the two genes obtained from previous studies [6].

In conclusion, this study suggests that the pharmacogenetic approach to DOACs is a relatively new field of research and that all observations made so far need further consideration and the execution of additional and larger replication studies. In this regard, a recent retrospective study performed on 1806 patients treated with dabigatran, rivaroxaban, or apixaban has identified and confirmed a close correlation between *ABCB1* single nucleotide variants and haplotypes with clinical outcomes in rivaroxaban and apixaban users [13]. Similarly, other recent studies have confirmed the correlation between *ABCB1* and *CES1* SNPs and plasma levels of dabigatran in Chinese patient populations [14]. Recent additional reviews of literature do not add much to what has been previously described [15,16].

In fact, due to the few case-control studies carried out, there are still insufficient clinical indications to suggest a valid use of these polymorphisms, as in the PharmGKB database (www.pharmgkb.org, accessed on 11 December 2021) where indications for SNPs recognized to date are indicated as “Level 3”, with a low level of evidence supporting the association [17].

In conclusion, the results obtained to date certainly suggest an incontrovertible influence of several sequence variants on the pharmacokinetics and the clinical outcomes of DOACs. However, the path towards the practical use of pharmacogenetics in this field is still to be determined, and the achievement of a clear genetic indication of response to treatment is not simple and requires further extensive research.

## Data Availability

Not applicable.
